# Metabolic Reprogramming of Mouse Bone Marrow Derived Macrophages Following Erythrophagocytosis

**DOI:** 10.3389/fphys.2020.00396

**Published:** 2020-04-30

**Authors:** Alexis Catala, Lyla A. Youssef, Julie A. Reisz, Monika Dzieciatkowska, Nicholas E. Powers, Carlo Marchetti, Matthew Karafin, James C. Zimring, Krystalyn E. Hudson, Kirk C. Hansen, Steven L. Spitalnik, Angelo D’Alessandro

**Affiliations:** ^1^Department of Biochemistry and Molecular Genetics, University of Colorado Denver – Anschutz Medical Campus, Aurora, CO, United States; ^2^Program in Structural Biology and Biochemistry, University of Colorado Denver – Anschutz Medical Campus, Aurora, CO, United States; ^3^Department of Microbiology and Immunology, Columbia University, New York, NY, United States; ^4^Department of Medicine – Division of Infectious Diseases, University of Colorado Denver – Anschutz Medical Campus, Aurora, CO, United States; ^5^Medical Sciences Institute, Blood Center of Wisconsin (Versiti), Milwaukee, WI, United States; ^6^Department of Pathology, University of Virginia, Charlottesville, VA, United States; ^7^Department of Pathology and Cell Biology, Columbia University, New York, NY, United States; ^8^Department of Medicine – Division of Hematology, University of Colorado Denver – Anschutz Medical Campus, Aurora, CO, United States

**Keywords:** macrophage metabolism, omics technologies, blood, OXPHOS, mitochondrial dysregulation, pentose phosphate pathway, lipid accumulation

## Abstract

Reticuloendothelial macrophages engulf ∼0.2 trillion senescent erythrocytes daily in a process called erythrophagocytosis (EP). This critical mechanism preserves systemic heme-iron homeostasis by regulating red blood cell (RBC) catabolism and iron recycling. Although extensive work has demonstrated the various effects on macrophage metabolic reprogramming by stimulation with proinflammatory cytokines, little is known about the impact of EP on the macrophage metabolome and proteome. Thus, we performed mass spectrometry-based metabolomics and proteomics analyses of mouse bone marrow-derived macrophages (BMDMs) before and after EP of IgG-coated RBCs. Further, metabolomics was performed on BMDMs incubated with free IgG to ensure that changes to macrophage metabolism were due to opsonized RBCs and not to free IgG binding. Uniformly labeled tracing experiments were conducted on BMDMs in the presence and absence of IgG-coated RBCs to assess the flux of glucose through the pentose phosphate pathway (PPP). In this study, we demonstrate that EP significantly alters amino acid and fatty acid metabolism, the Krebs cycle, OXPHOS, and arachidonate-linoleate metabolism. Increases in levels of amino acids, lipids and oxylipins, heme products, and RBC-derived proteins are noted in BMDMs following EP. Tracing experiments with U-^13^C_6_ glucose indicated a slower flux through glycolysis and enhanced PPP activation. Notably, we show that it is fueled by glucose derived from the macrophages themselves or from the extracellular media prior to EP, but not from opsonized RBCs. The PPP-derived NADPH can then fuel the oxidative burst, leading to the generation of reactive oxygen species necessary to promote digestion of phagocytosed RBC proteins via radical attack. Results were confirmed by redox proteomics experiments, demonstrating the oxidation of Cys152 and Cys94 of glyceraldehyde 3-phosphate dehydrogenase (GAPDH) and hemoglobin-β, respectively. Significant increases in early Krebs cycle and C_5_-branched dibasic acid metabolites (α-ketoglutarate and 2-hydroxyglutarate, respectively) indicate that EP promotes the dysregulation of mitochondrial metabolism. Lastly, EP stimulated aminolevulinic acid (ALA) synthase and arginase activity as indicated by significant accumulations of ALA and ornithine after IgG-mediated RBC ingestion. Importantly, EP-mediated metabolic reprogramming of BMDMs does not occur following exposure to IgG alone. In conclusion, we show that EP reprograms macrophage metabolism and modifies macrophage polarization.

## Introduction

Erythrocyte (red blood cell, RBC) function extends beyond the monumental task of maintaining systemic acid/base equilibria via oxygen, carbon dioxide, and the transport of nutrients to tissues. Other vital roles include erythrocrine function [i.e., release of metabolically active molecules such as adenosine triphosphate (ATP) and nitric oxide (NO)], redox regulation, systemic hemodynamics, immunomodulation, and iron metabolism (Alayash et al., 2001; Kuhn et al., 2017). In adults, RBCs account for ∼83% of the total cells in the body (25 out of ∼30 trillion) and have an average lifespan of 120 days; thus, ∼0.2 trillion RBCs are cleared from the bloodstream and generated *de novo* on a daily basis (Nemkov et al., 2018). Importantly, RBC damage and changes to deformability are directly linked to several severe pathologies (Caspary et al., 1967; Yoshida et al., 2019) including endothelial dysfunction (Kuhn et al., 2017), anemia (Alsultan et al., 2010; Belanger et al., 2015; Belcher et al., 2017), sepsis (Larsen et al., 2010), diabetic nephropathy (Brown et al., 2005), and thrombosis (Barr et al., 2013; Weisel and Litvinov, 2019).

Recycling of iron derived from RBCs is essential for sustaining erythropoiesis. As much as 70% of the total iron in the human body, or 3–5 g, is contained within RBCs, specifically in the heme protoporphyrin rings of hemoglobin (a single RBC contains ∼1.0 billion heme moieties per ∼250 million hemoglobin molecules; Gkouvatsos et al., 2012; Korolnek and Hamza, 2015; Yoshida et al., 2019). Notably, iron is a potent catalyst for generating reactive oxygen species (ROS) via the Fenton reaction, which can quickly lead to systemic toxicity due to the high reactivity of iron when free in the circulation (e.g., upon overload of transferrin, the plasma iron chaperone) (Papanikolaou and Pantopoulos, 2005; Kosman, 2010; Hod et al., 2010; Korolnek and Hamza, 2015; Rapido et al., 2017; Youssef and Spitalnik, 2017a). For this reason, highly specialized mechanisms are required for regulating RBC catabolism and iron recycling. To this end, macrophages are integral to the tight regulatory mechanism of RBC clearance (de Back et al., 2014; Klei et al., 2017) Reticuloendothelial macrophages (REMs), primarily in the spleen and liver, opsonize senescent RBCs in a process called erythrophagocytosis (EP) (Gkouvatsos et al., 2012; de Back et al., 2014).

With ∼2 million RBCs being recycled every second via this mechanism, EP is the largest source of iron flux in the body (Korolnek and Hamza, 2015). Excessive EP by individual macrophages can lead to ferroptosis both *in vitro* and *in vivo* (Dixon et al., 2012; Youssef and Spitalnik, 2017a). This form of iron-induced, non-apoptotic cell death is characterized by an overwhelming, iron-dependent accumulation of lethal ROS derived from lipid peroxidation (Dixon et al., 2012; Cao and Dixon, 2016). During this process, free radicals can strip electrons from unsaturated fatty acid components of membrane lipids, initiating a self-propagating chain reaction and massive oxidative destruction of lipids (Yang and Stockwell, 2016; Ramana et al., 2017). A bolus of intracellular iron and heme due to EP can also upregulate transcription of aminolevulinic acid (ALA) synthase, using glycine and succinyl-CoA from the Krebs cycle to produce ALA and initiate porphyrin (the heme precursor) synthesis. Other heme-responsive genes include heme oxygenase 1 (HO-1), a heme-catabolizing, and anti-inflammatory enzyme associated with maintaining the integrity of the REM lineage (Kovtunovych et al., 2010; Naito et al., 2014; Soares and Hamza, 2016), and SPI-C, a E26 transformation-specific (Ets) transcription factor required for the development of splenic and bone marrow (F4/80^hi^) macrophages (Kohyama et al., 2009; Haldar et al., 2014). In the clinic, hypoferremia (iron-deficiency) and heme-catabolizing enzyme deficiencies (e.g., HO-1 deficiency) can cause progressive depletion of erythrophagocytic macrophage populations, profoundly deregulating heme-iron metabolism and homeostasis (Guida et al., 2015; Soares and Hamza, 2016).

Two major macrophage subsets (M1/M2) were identified, based on their polarization status, and defined by their different functional programs, migration mode, and cytokine secretion profiles (Labonte et al., 2014; Curi et al., 2017; Mould et al., 2017; Remmerie and Scott, 2018). Classically activated (M1) macrophages are described as proinflammatory macrophages that are more migratory in nature and release inflammatory cytokines, such as interleukin-6 (IL-6), interferon-γ (IFN-γ), and tumor necrosis factor-α (TNF-α), to aid in the amplification of immune responses and directed microenvironmental remodeling (Martinez and Gordon, 2014; Meiser et al., 2016; Gaber et al., 2017; Nonnenmacher and Hiller, 2018). By contrast, alternatively activated (M2) macrophages are typically less migratory owning to their extensive roles in repairing tissue-damage, and have a proresolution profile characterized by the secretion of cytokines, such as transforming growth factor-β (TGF-β), IL-4, IL-10, and IL-13 (Zhang and An, 2007; Nahrendorf and Swirski, 2013; Yang and Ming, 2014). Although the M1/M2 polarization nomenclature is helpful in binning macrophage subsets, macrophage populations are actually quite heterogenous, polarization is both transient and plastic, intermediate forms have been seen, and other subtypes have been proposed (e.g., M4, Mox, Mhem) (Sica and Mantovani, 2012; Nahrendorf and Swirski, 2013; Labonte et al., 2014; Youssef and Spitalnik, 2017b; Viola et al., 2019). The focus of the studies described herein will be on the metabolic differences between these subsets and not their cytokine profiles.

M1 macrophages exhibit a metabolic shift similar to the cancer-associated Warburg effect, in which the Krebs cycle has been altered and there are increases in lactate production and an amplified flux through the pentose phosphate pathway (PPP) (Kelly and O’Neill, 2015; Viola et al., 2019). Importantly, amplified PPP flux fuels the production of reduced nicotinamide adenine dinucleotide phosphate (NADPH), thereby promoting ROS generation via NADPH oxidase (Kelly and O’Neill, 2015). Increases in mitochondrial ROS can negatively affect Complex II activity in the electron transport chain, prompting accumulation of succinate (Zhao et al., 2019). Succinate can then stabilize hypoxia-inducible factor 1-alpha (HIF-1α) via inhibited hydroxylation, inducing expression of glycolytic enzymes and proinflammatory IL-1β (Tannahill et al., 2013). M1 macrophages also have increased phenylalanine production, which promotes the expression and activity of GTP cyclohydrolase I (Li et al., 2007). This allows for directed modulation of tetrahydrobiopterin synthesis and, by extension, nitric oxide (NO) production (Li et al., 2007). In contrast, M2 macrophages are not as dependent on glycolysis for producing energy-fueling metabolites like ATP and, instead, rely more heavily on mitochondrial metabolism (Kelly and O’Neill, 2015; Noe and Mitchell, 2019; Viola et al., 2019). Their polarization state is partly mediated by increases in intracellular itaconate (an anti-inflammatory metabolite that activates Nrf2 via KEAP1 alkylation) (Mills et al., 2018; O’Neill and Artyomov, 2019) and arginine metabolism (Noe and Mitchell, 2019). Arginine is preferentially metabolized to produce ornithine and urea instead of NO via arginase 1 (Arg-1) to promote polyamine generation and HIF1α destabilization (Kelly and O’Neill, 2015; Noe and Mitchell, 2019).

Fundamental to macrophage activity, metabolic reprogramming modulates key immune functions such as phagocytosis, inflammatory cytokine signaling, and extracellular matrix remodeling (Curi et al., 2017; Van den Bossche et al., 2017; Nonnenmacher and Hiller, 2018; D’Alessandro et al., 2018). Despite extensive evidence highlighting the central role of metabolic reprogramming following stimulation with proinflammatory factors (e.g., LPS, INF-γ) (Martinez and Gordon, 2014; Kelly and O’Neill, 2015), there is limited information that directly addresses the impact of phagocytosis itself on macrophage metabolism. To the best of our knowledge, no study has yet focused on these aspects within the framework of EP. Therefore, we addressed this deficit by conducing metabolomic analyses on bone marrow derived macrophages (BMDMs) that had phagocytosed IgG-opsonized RBCs. Given the metabolic derangement that can ensue due to heme-iron dysregulation, we hypothesize BMDMs that have engulfed iron-loaded RBCs will be burdened by increased oxidant stress and accumulate eicosanoids and oxylipins.

## Materials and Methods

### Mice

Wild-type C57BL/6 mice (6–12 weeks of age) were purchased from Jackson Laboratories. All procedures were approved by the Institutional Animal Care and Use Committee at Columbia University (New York City, NY, United States). Each experiment was conducted at least twice in biological triplicates (*n* = 3 per group, each independent experiment).

### RBC Collection

Mice were bled aseptically via cardiac puncture and pooled whole blood was placed in CPDA-1 solution obtained from human primary collection packs (Baxter International, Deerfield, IL, United States) to a final concentration of 15% CPDA-1. Whole blood was log4 leukofiltered using Neonatal High-efficiency Leukocyte Reduction Filters (Purecell Neo; Pall Corporation, Port Washington, NY, United States). RBCs from leukofiltered blood were packed by centrifugation (10 min, 4°C, 1,000 × *g*) and a portion of the plasma-containing CPDA-1 supernatant was removed to yield a hematocrit of ∼75%. Packed RBCs were used “fresh” (i.e., stored < 24 h at 4°C) to interrogate EP.

### *In vitro* EP Assay

Mononuclear cells obtained from mouse femurs were cultured for 7–11 days in Iscove’s Modified Dulbecco’s Medium supplemented with 10% FBS, 2 mM L-glutamine, 50 units/ml penicillin, 50 μg/mL streptomycin, 20 μg/mL gentamycin (Thermo Fisher Scientific, Waltham, MA, United States) and 20 ng/mL human macrophage colony-stimulating factor (M-CSF) (PeproTech, Rocky Hill, NJ, United States) to generate primary BMDMs. Opsonized RBCs were generated by incubating RBCs with rabbit, anti-mouse RBC IgG at 0.5 mg/mL (Rockland Immunochemicals, Limerick, PA, United States). Adherent BMDMs were plated 24 h prior to the experiment and incubated with PBS or opsonized RBCs for 2 h at 37°C. Following incubation, aliquots of the extracellular media were taken for metabolomic analysis of supernatants and non-ingested RBCs were lysed. Adherent BMDMs were washed with PBS, collected by scraping, and pelleted for metabolic analysis of cell pellets.

### *In vitro* IgG Assay

Mononuclear cells obtained from bone marrow of C57BL/6J mice were seeded at 1 × 10^6^ cell/mL in a 48-well plate and differentiated for 7 days in RPMI 1,640 medium supplemented with 10% FCS, 1% penicillin/streptomycin (P/S) and 10 ng/mL M-CSF (R&D system; Minneapolis, MN, United States). Cells were then washed with (1x) PBS, and complete media in the absence of M-CSF was added to the cultures. Vehicle or mouse IgG (0.5 mg/mL) (R&D System, Minneapolis, MN, United States) were added for 1 h at 37°C. Following incubation, cells were washed and stored at –80°C for metabolomics. Thawed cells were lysed by adding lysis buffer directly to wells and removed by scraping.

### Metabolic Labeling Experiments

Uniformly labeled ^13^C_6_-glucose (Cambridge Isotope Laboratories, Tewksbury, MA, United States) was incubated with RBCs (161 mM for 50% final ratio of labeled glucose) for 4 h at 37°C, before being gently washed with PBS to remove extracellular ^13^C_6_-glucose. For labeling BMDMs, ^13^C_6_-glucose was supplemented in the media used for culturing BMDMs (25 mM for 50% final ratio of labeled glucose) 1 h prior to, or during, EP. Samples were collected at 1, 3, and 12 h, and metabolites from cells extracted (in the absence of heavy labeled internal standards) for metabolomic analysis.

### UHPLC-MS Metabolomics

Metabolites were extracted from cell pellets (∼1 × 10^6^ cells) or supernatants at a dilution of 1:10 or 1:25, respectively, in ice-cold lysis buffer (5:3:2 MeOH:ACN:H_2_O) in the presence of a mix of heavy labeled internal standards. These standards included 15 uniformly labeled ^13^C^15^N-amino acids at a final concentration of 2.5 μM, ^13^C_1_-lactate (40 μM), ^13^C_5_-2-α-ketoglutarate,^13^C_4_-succinate, ^13^C_1_,_4_-fumarate, 2H_4_-prostaglandin E2, and 2H_8_-arachidonic acid at a final concentration of 1 μM (Cambridge Isotopes Laboratories, Inc., Tewksbury, MA, United States). Samples were vortexed, insoluble material pelleted, and supernatants collected. Extracts (20 μL) were injected into a Thermo Vanquish UHPLC system coupled to a Thermo Q Exactive mass spectrometer (Vanquish – Q Exactive; Thermo Fisher Scientific, San Jose, CA, United States and Bremen, Germany) with electrospray ionization for metabolomic analysis, as described (Catala et al., 2018). Technical mixes were generated by pooling aliquots of extracts and ran every 3 analytical runs to control for technical variability as judged by coefficients of variation (CVs). Metabolite assignments, isotopologue distributions, and correction for expected natural abundancies of ^13^C were performed using MAVEN (Princeton, NJ, United States) (Melamud et al., 2010). Discovery mode analysis was performed with standard workflows using Compound Discoverer (Thermo Fisher Scientific, Waltham, MA, United States).

### UHPLC-MS Lipidomics

Hydrophobic metabolites were extracted from cell pellets (∼1 × 10^6^ cells) in ice-cold methanol at a 1:10 dilution. Samples were quickly vortexed at room temperature followed by incubation at -20°C for 30 min and centrifugation (18,213 × *g*, 10 min, 4°C). Supernatants (40 μL) were diluted 1:1 using 10 mM ammonium acetate and protein pellets stored at −80°C. Extracts (20 μL) were injected into our UHPLC-MS system with electrospray ionization. Metabolites were separated on a 150 × 2.1 mm, 1.8 μm Acquity HSS T3 column (Waters, Milford, MA, United States) at 45°C using a 17 min gradient method at 300 μL/min and mobile phases (A: 75:25 H_2_O:ACN, 5 mM NH_4_OAc; B: 50:45:5 IPA:ACN:H_2_O, 5 mM NH_4_OAc) for negative ion mode. Solvent gradient: 0–1.0 min 25% B; 1.0–2.0 min 50% B; 2.0–8.0 min 90% B; 8.0–10.0 min 99% B; 10.0–14.0 min hold at 99% B; 14.0–14.1 min 25% B, 400 μL/min; 14.1–16.9 min hold at 25% B, 400 μL/min; 16.9–17.0 min hold at 25% B. Technical mixes were generated, ran and technical variability assessed, as described above.

### Nano-UHPLC-Tandem MS Proteomics

Proteins extracted from BMDM cell pellets were separated by SDS-PAGE, and bands ranging from ∼20 to 80 kDa were excised, reduced, alkylated, and trypsin digested. Extracted peptides were analyzed by nanoLCMS-MS (Thermo EASY-nLC 1,000 – Q Exactive HF; Thermo Fisher Scientific; San Jose, CA, United States and Bremen, Germany) and separated on a house-made 15 cm C18 analytical column (100 mm inner diameter) packed with Cortecs C18 resin (2.7 mm; Phenomenex, Torrance, CA, United States), using a 80 min linear gradient of 2–32% ACN at 350 nL/min.

### Statistical Analysis

Statistical (i.e., *t*-test, ANOVA, linear regression and Spearman correlations) and multivariate [i.e., principal component analysis (PCA), partial least squares-discriminant analysis (PLS-DA), and hierarchical clustering analysis (HCA)] analyses, heat maps, and graphs were performed and prepared using GraphPad Prism 5.0 (GraphPad Software, Inc., La Jolla, CA, United States), Morpheus (Broad Institute; Boston, MA, United States), and MetaboAnalyst 4.0 (Chong et al., 2018).

## Results

### EP Induces Extensive Metabolic Reprogramming of BMDMs

**FIGURE 1:**
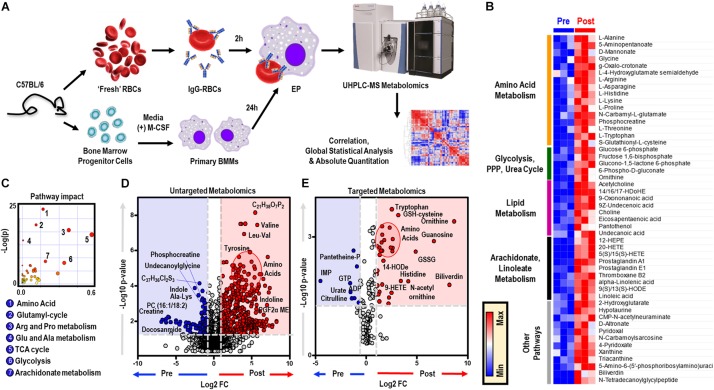
*In vitro* assessment of BMDM EP. Mouse RBCs were incubated with rabbit, anti-mouse RBC IgG. BMDMs were then incubated with PBS (control) or with IgG-coated RBCs for *in vitro* opsonization to reflect EP. **(A)** Metabolic correlates identified before (Pre) and after (Post) EP were plotted as a hierarchically-clustered heat map **(B)**. The metabolome view map of relevant metabolic pathways showed significant changes in cellular metabolic pathways following EP **(C)**. Univariate analysis of the BMDM metabolome using untargeted **(D)** and targeted **(E)** metabolomics methods to identify metabolites that change due to EP. The region highlighted in red (fold change (FC) ≥ 2.0; *p*-value < 0.05) indicates metabolites that are present in significantly higher amounts in BMDMs following EP (Post); whereas, the region highlighted in blue (fold change ≤ 0.5; *p*-value < 0.05) indicates metabolites that accumulate in BMDMs before EP (Pre). Amino acids identified clustered in similar regions of Pre were encircled (*red*).

To elucidate the metabolic phenotype of macrophages following EP, BMDMs were isolated from C57BL/6 mice and incubated with IgG-opsonized RBCs for 2 h at 37°C. Extracellular media (supernatants) and cell pellets were collected for metabolomics (Figure 1A and analyzed using untargeted (Figure 1D) and targeted (Figure 1E) mass spectrometry methods. In Supplementary Figures S1A,B, we provide an overview of the untargeted metabolomics workflow and volcano plot from the data generated through this approach; in addition, we report absolute quantification in cells and supernatants are provided in Supplementary Figures S1C,D for of amino acids, and Supplementary Figures S1E,F glycolytic metabolites and oxylipins. An extensive report of the metabolomics data is provided in [Supplementary Table S1 and Supplementary Figures S1A,B, *Untargeted, Targeted (5 MM), and Global (5 MM) Tabs*]. Similarly, cell pellet metabolomics analyses were performed on BMDMs incubated with IgG to ensure that changes to BMDM metabolome were due to ingestion of IgG-opsonized RBCs and not to IgG binding alone (Supplementary Figure S2 and extensively reported in Supplementary Table S2). Increased levels of tryptophan, flavin mononucleotide (FMN, *p* = 0.0002), maltose (*p* = 0.0002), and 2-methyleneglutarate (2-MG, *p* = 0.0021) were noted in BMDMs that were incubated with IgG and are indicative of reprogramming of riboflavin, sugar, and C_5_-dibasic acid metabolism (*data not shown*). Although this could have implications for macrophage effector function, similar to what has been reported for dendritic cell activation by Toll-like receptor signaling (Thwe et al., 2017), these results will not be discussed further except in the context of comparing particular metabolites found to be involved in EP-mediated metabolic reprogramming. Following EP, BMDMs were characterized by substantial increases in hundreds of metabolites (Figures 1D,E), some of which are plotted in the heat maps of Figures 1B, 5A. Specifically, enrichment was observed in pathways involved in amino acid and fatty acid metabolism, the Krebs cycle [also referred to as the tricarboxylate acid (TCA) cycle], and arachidonate metabolism (pathway leading to eicosanoid and oxylipin production; Figure 1C).

### EP Increases Metabolites Involved in PPP and GSH Metabolism

**FIGURE 2:**
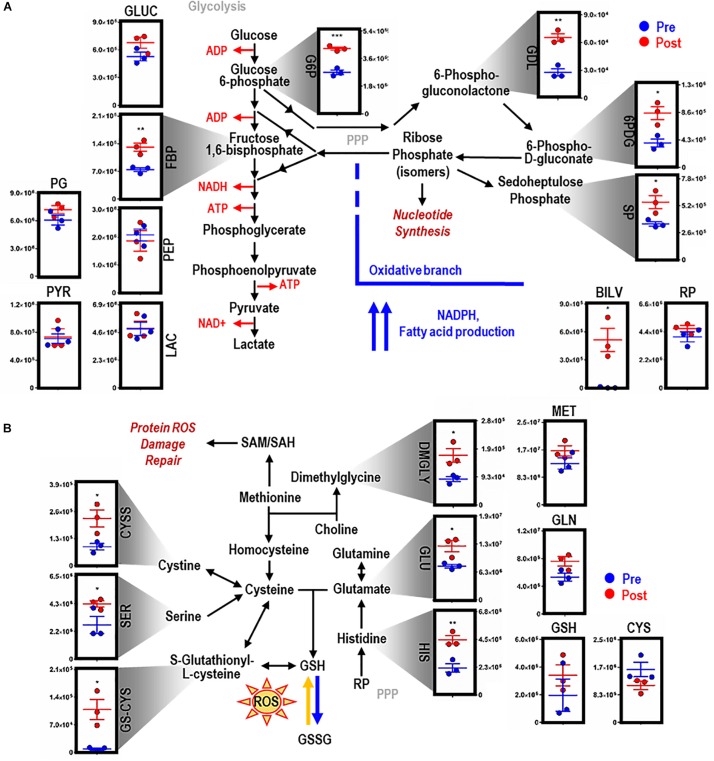
EP reprograms glycolysis, the pentose phosphate pathway, and glutathione metabolism. Metabolites from glycolysis and the pentose phosphate pathway (PPP) **(A)**. As part of heme metabolism, biliverdin (BILV) confirms successful EP of IgG-opsonized RBCs by BMDMs. Entry into glutathione (GSH) metabolism via methionine (MET) and glutamate (GLU) **(B)**. For all plots, the y-axis represents relative intensity (a.u.). *p ≤ 0.05; **p ≤ 0.01; ***p ≤ 0.001 (unpaired *t*-test, 2-tailed distribution). GLUC, glucose; G6P, glucose 6-phosphate; FBP, fructose bisphosphate; PG, phophoglycerate; PEP, phosphoenolpyruvate; PYR, pyruvate; LAC, lactate; GDL, 6-phosphate gluconolactone; 6PDG, 6-phospho-D-gluconate; SP, sedoheptulose phosphate; RP, ribose phosphate (isomers); GLN, glutamine; CYS, cysteine; CYSS, cystine; SER, serine; GS-CYS, S-gultathionyl-L-cysteine; HIS, histidine; DMGLY, dimethylglygine; SAM/H, S-adenosyl methionine/homocysteine.

**FIGURE 3:**
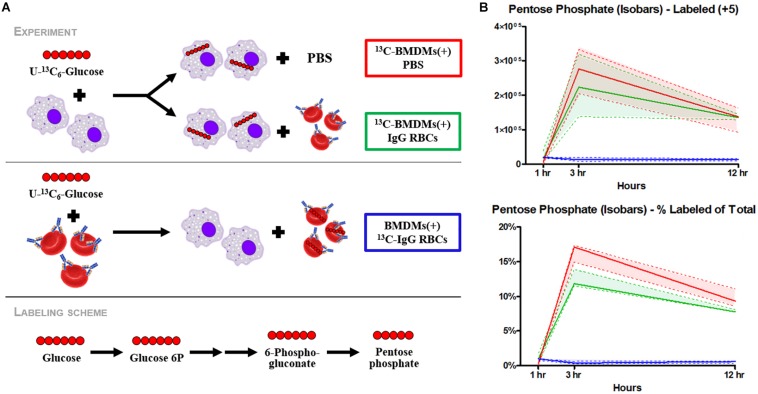
Flux analysis of BMDMs incubated with heavy labeled substrates. BMDMs in PBS (*red*) or with IgG-opsonized RBCs (*green*) were uniformly labeled with ^13^C_6_-glucose **(A)**. Experimental results of ^13^C_6_-glucose detection in BMDMs **(B)**. Enrichment of the + 5 isotopologues of PPP metabolites, such as pentose phosphate isobaric isomers, were observed (*top*). Total percent labeled amounts of these isomers are shown in the bottom panel. These carbons are derived from glucose flux from the Embden-Meyerhof glycolytic pathway through the PPP. Continuous lines reflect the median of the three groups; whereas, the dashed lines represent interquartile ranges.

EP promoted significant accumulation of early [i.e., glucose 6-phosphate (G6P), *p* = 0.009; fructose bisphosphate (FBP), *p* = 0.0078) but not late (e.g., pyruvate (PYR), lactate (LAC)] glycolytic metabolites (Figure 2A). Additionally, following EP, intermediates of the oxidative [i.e., 6-phosphogluconolactone (GDL), *p* = 0.0027; 6-phosphogluconate (6PDG), *p* = 0.0170] and non-oxidative [i.e., sedoheptulose phosphate (SP), *p* = 0.0109] branches of the PPP increased significantly (Figure 2A). These results suggested a slower flux through glycolysis and enhanced PPP activation. To test this hypothesis, tracing experiments were performed by incubating BMDMs with ^13^C_6_-glucose 1 h prior to and during EP (Figure 3). These results indicated that ^13^C_5_-ribose phosphate and pentose phosphate isobaric isomers were exclusively labeled from glucose taken up *de novo* during EP (Figure 3B, *green*) or from endogenous glucose already in the macrophage prior to EP (Figure 3B, *red*), but not from the RBCs themselves (Figure 3B, *blue*). This suggests that RBC-derived cytosolic sugars do not participate in fueling this activation of glycolysis or the PPP. PPP activation is consistent with (i) increased oxidant stress resulting from phagocytosis of iron-loaded RBCs; and (ii) increased flux through NADPH-generating pathways to fuel NADPH oxidase-dependent ROS production to target the engulfed cell via radical attack (Xu et al., 2016). Consistent with increased oxidant stress, decreases in free reduced cysteine and significant increases in oxidized cysteine disulfide [cystine (CYSS), *p* = 0.0348] and cysteine glutathione disulfide [S-glutathionyl-L-cysteine (GS-CYS), *p* = 0.0110] were noted (Figure 2B). Further, higher levels of histidine (HIS, *p* = 0.0091), glutamate (GLU, *p* = 0.0283), methionine, serine (SER, *p* = 0.0392), and dimethylglycine (DMGLY, *p* = 0.0308) indicate alterations of glutathione and sulfur homeostasis and one-carbon metabolism, which are involved in repairing oxidant protein damage (Reisz et al., 2018; Figure 2B and Supplementary Figure S1C). Importantly, these metabolites did not increase when BMDMs were incubated with IgG alone (the subset of significant metabolites with pathways noted in Supplementary Figures S2C,D).

### EP Promotes Purine Oxidation and Dysregulation of Mitochondrial Metabolism

**FIGURE 4:**
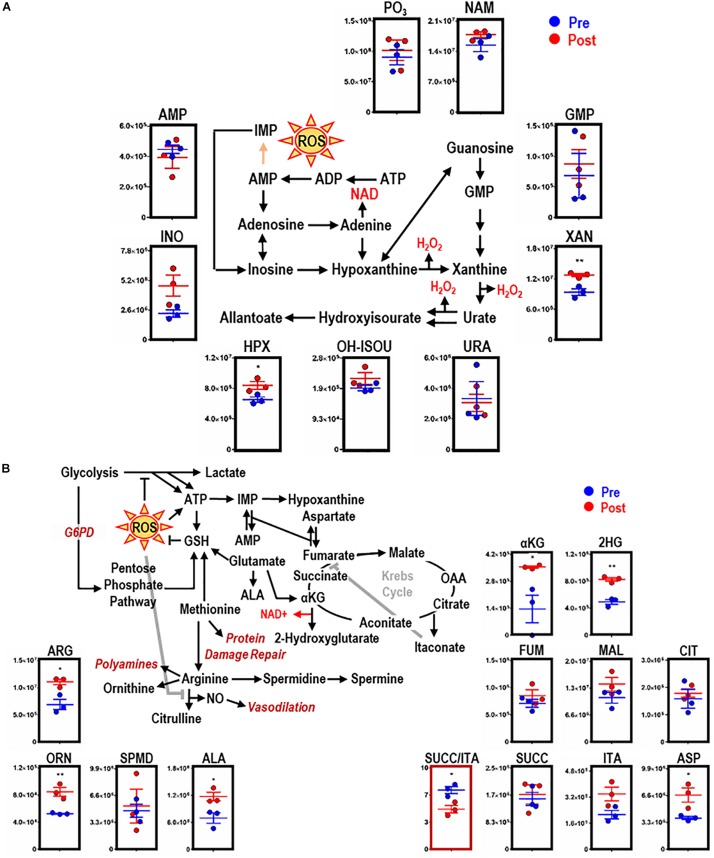
EP remodels purine and arginine metabolism, glutathione homeostasis, and the Krebs cycle in BMDMs. Overview and downstream metabolites of purine metabolism with entry into this pathway beginning with adenosine triphosphate (ATP) **(A)**. Overview of arginine (ARG) metabolism, GSH homeostasis, and the Krebs cycle **(B)**. For all plots, the y-axis represents relative intensity (a.u.). *p ≤ 0.05; **p ≤ 0.01; ***p ≤ 0.001 (unpaired *t*-test, 2-tailed distribution). PO_3_, phosphate; URA, urate; AMP, adenosine monophosphate; NAM, nicotinamide; HPX, hypoxanthine; INO, inosine; XAN, xanthine; ALA, aminolevulinate; ASP, aspartate; 2HG, 2-hydroxyglutarate; SUCC, succinate; ORN, ornithine; OH-ISOU, hydroxyisourate; SPMD, spermidine; SPM, spermine; ITA, itaconate; FUM, fumarate; CIT, citrate; MAL, malate; αKG, α-ketoglutarate.

Consistent with dysregulated glutathione homeostasis, increased purine oxidation was observed in macrophages upon EP, as indicated by ATP breakdown and increases in oxidation products, including inosine, hypoxanthine (HPX, *p* = 0.0377), and xanthine (XAN, *p* = 0.0088) (Figure 4A). HPX and XAN did not accumulate upon incubation of BMDMs with IgG alone (Supplementary Figure S2C). Purine oxidation is tied to mitochondrial metabolism via salvage reactions fueled by aspartate consumption and fumarate generation. Of note, higher levels of intracellular aspartate (ASP, *p* = 0.0332), but not fumarate, were observed in macrophages upon EP. Further, increases in α-ketoglutarate (αKG, *p* = 0.0048), 2-hydroxyglutarate (2HG, *p* = 0.0017), and itaconate were detected after EP in the absence of increases in late carboxylic acid intermediates (e.g., succinate, fumarate; Figure 4B). Hence, significant decreases in succinate to itaconate ratios (SUCC/ITA, *p* = 0.0215) were noted (Figure 4B, *boxed in red*). Consistent with decreased NO synthase (NOS) activity and increased arginase activity following EP, increases in free arginine (ARG, *p* = 0.0173) and ornithine (ORN, *p* = 0.0074), but not citrulline or polyamines (spermidine), were noted (Figure 4B). Lastly, upregulation of ALA synthase, due to an intracellular bolus of iron and heme in BMDMs during EP, was confirmed by a significant increase of ALA (*p* = 0.0344; Figure 4B). Increased levels of these metabolites were not observed when BMDMs were incubated with IgG alone (Supplementary Figure S2E, *subset of significant metabolites*).

### EP Induces Significant Increases in Prostaglandins and Oxylipins

**FIGURE 5:**
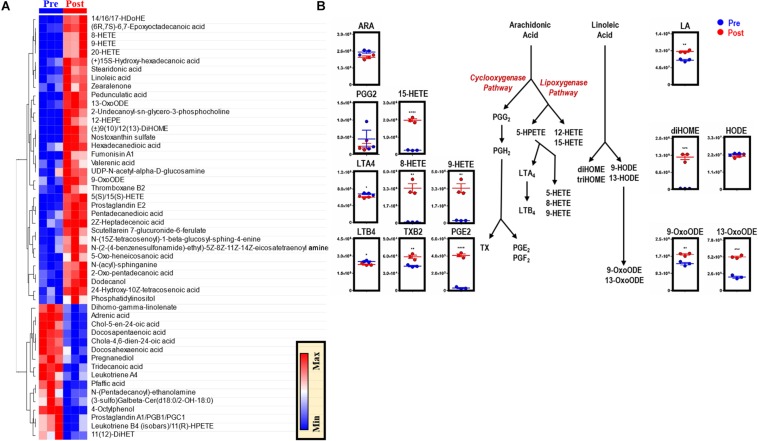
EP reprograms arachidonate and linoleate metabolism in BMDMs. The top 50 metabolites in BMDMs that are part of eicosanoid and oxylipin synthesis and change significantly following EP **(A)**. Values are plotted as a hierarchically-clustered heat map based on *p*-value. Overview of eicosanoid and oxylipin metabolism, stemming from arachidonic acid (ARA) and linoleic acid (LA) **(B)**. For all plots, the y-axis represents relative intensity (a.u.). *p ≤ 0.05; **p ≤ 0.01; ***p ≤ 0.001 (unpaired *t*-test, 2-tailed distribution). PGG2, prostaglandin G2; PGH2, prostaglandin H2; PGE2, prostaglandin E2; PGF2, prostaglandin F2; HETE, hydroxyeicosatetraenoic acid; HPETE, hydroperoxyeicosatetraenoic acid; LTA4, leukotriene A4; LTB4, leukotriene B4; TX, thromboxane; TXB2, thromboxane B2; diHOME, dihydroxyoctadecanoic acids; triHOME, trihydroxyoctadecanoic acids; HODE, hydroxyoctadecadienoic acid; OxoODE, oxooctadecadienoic acid.

Phospholipid hydrolysis generates free fatty acids, including arachidonate (the direct precursor of eicosanoids), thereby directly modulating inflammation, immunity, and other signaling pathways (Esser-von Bieren, 2017). Dysregulation of intracellular redox metabolism can result in increased peroxidation of macrophage membrane-derived lipids. Following EP, macrophages are exposed to a bolus of RBC membrane-derived lipids that are susceptible to lipid peroxidation and iron stores that can activate cyclooxygenases (COXs) and generate proinflammatory eicosanoid derivatives (Youssef et al., 2018). Therefore, lipidomics analyses were performed to investigate the production of eicosanoids and oxylipins in BMDMs after EP [Figure 5 and extensively reported in Supplementary Table S1, *Global (17MM) Tab*]. Decreases in arachidonic acid were found, accompanied by decreases in leukotrienes and significant increases in thromboxane B2 (TXB2, *p* = 0.0070), prostaglandin E2 (PGE2, *p* < 0.0001), and hydroxyeicosatrienate (HETE) derivatives [5(S)/15(S)-HETE, *p* < 0.0001; 8-HETE, *p* = 0.0016; 9/20-HETE, *p* = 0.0019] (Figure 5). Further, increases in linoleic acid (LA, *p* = 0.0013) were accompanied by accumulation of dihydroxyoctadecenate (diHOME, *p* = 0.0003) and oxooctadecadienates (9-oxoODE, *p* = 0.0032; 13-oxoODE, *p* = 0.0001), without increases in hydroxyoctadecadienate (HODE) (Figure 5).

### EP Modulates the Protein Landscape, Including Modifications of Key Metabolic Enzymes

**FIGURE 6:**
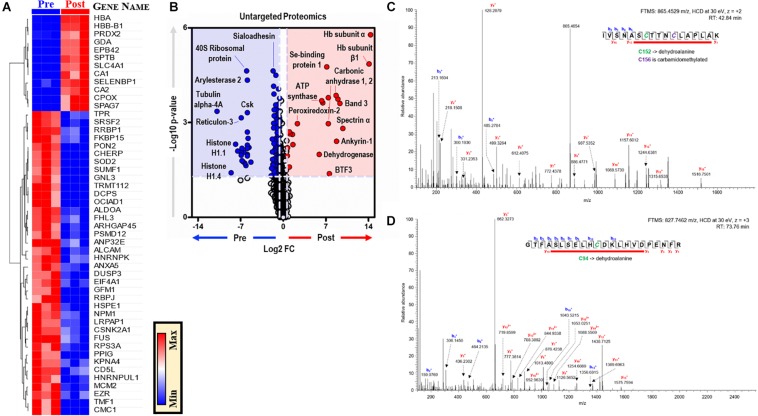
EP alters the BMDM proteome. **(A)**. The top 50 proteins in BMDMs that are significantly changed by EP **(A)**. Values are plotted as a hierarchically-clustered heat map based on *p*-value with proteins listed by gene name. Univariate analysis of the BMDM proteome using untargeted proteomics to identify proteins that change due to EP **(B)**. The region highlighted in red (FC > 1.5; *p* < 0.05) indicates proteins present in significantly higher amounts in BMDMs after EP (Post); whereas, the region highlighted in blue (FC < 0.67; *p* < 0.05) indicates proteins found to be accumulated in BMDMs before EP (Pre). Representative tandem mass spectrometry spectra with deduced protein sequences for glyceraldehyde 3-phosphate dehydrogenase (GAPDH) **(C)** and hemoglobin-β **(D)**. Dehydroalanine modification of Cys152 and carbamidomethylation of Cys156 in the active site of GAPDH **(C)**. Dehydroalanine modification of Cys94 adjacent to the heme binding site (H93, *proximal*) in the hemoglobin-β chain **(D)**.

Proteomics analyses of BMDMs, before and after EP, identified ∼3,000 unique proteins (Supplementary Table S3). Unsupervised analyses, including PLS-DA, identified significant effects of EP on the BMDM proteome, contributing to ∼51% of the total variance (Supplementary Figure S2A). Top proteins are presented in a PLS-DA variable importance in projection (VIP) plot (Supplementary Figures S3A,B) and in heat maps of Supplementary Figure S3C (*top 15*) and Figure 6A (*top 50*). EP induced increases in RBC-derived protein components (e.g., hemoglobin, band 3, spectrin, ankyrin, selenium-binding protein, peroxiredoxin 2) in BMDMs (Figure 6B). Notably, the functional cysteine (Cys) residues of RBC-derived proteins were irreversibly oxidized to dehydroalanine, including Cys94 of hemoglobin-β and Cys152 of glyceraldehyde 3-phosphate dehydrogenase (GAPDH) (Figures 6C,D). Conversely, cytoskeletal components (e.g., tubulin 4A), ribosomal proteins (e.g., 40S ribosomal protein), mitochondrial components, and nuclear components (e.g., histones) were decreased after EP (Figure 6B). No significant changes in interleukins, interferons, or interferon-dependent proteins were observed after EP. Notably, the terminal oxidase in mitochondrial electron transport (COX5A) increased significantly after EP, which is consistent with the alterations to mitochondrial metabolism discussed previously.

## Discussion

Metabolic reprogramming is critical to the immune response and contributes significantly to macrophage effector function (Martinez and Gordon, 2014). Macrophage immunometabolism is affected by various factors, including cytokines (Martinez and Gordon, 2014), complement components (Bohlson et al., 2014), pathogen-derived molecules (e.g., LPS) (Tannahill et al., 2013), and environmental stimuli [e.g., diet, Ji et al., 2012, and dietary antioxidants, such as pyrroloquinoline quinone (Van den Bossche et al., 2017; Friedman et al., 2018). Reprogramming of macrophage metabolism contributes to the etiopathogenesis of several diseases, including pulmonary hypertension (Pugliese et al., 2017; D’Alessandro et al., 2018), age-related chronically inflammatory diseases (Oishi and Manabe, 2016; Parisi et al., 2018), neurodegenerative diseases (Peruzzotti-Jametti et al., 2018), and inflammatory complications following ischemia and reperfusion (Chouchani et al., 2014; Huen and Cantley, 2015). However, limited knowledge is available about the impact of phagocytic activity and increased heme-iron burden on macrophage metabolism, which is crucial for pathogen defense mechanisms and general system homeostasis.

The present study investigated the impact of EP on macrophage metabolism and revealed that EP activates a series of pathways consistent with polarization toward the M2 metabolic phenotype, including those that support the generation of reducing cofactor NADPH. We address the question of whether these metabolic shifts were fueled by catabolism of RBC-derived substrates through glucose tracing experiments. Interestingly, EP fuels the PPP primarily by using glucose from extracellular sources or from macrophage reserves, rather than from RBC-derived sugar moieties. Whereas PPP activation in other non-phagocytic cell types typically counteracts oxidant stress, macrophages specifically use this pathway to produce ROS by, for example, upregulating nicotinamide adenine dinucleotide phosphate oxidase (NOX) 1 and 2 expression (Xu et al., 2016). Through increased NOX catalytic activity, superoxide generation enhances ROS production by macrophages, thereby supporting phagocytic activity during microbial infections and the use of heme-iron for electron transport across biological membranes (Xu et al., 2016). Further, NOX-generated ROS increase proteolytic attack of phagocytosed cellular components, which correlates with the redox proteomics analysis results presented herein. Although we expected to observe significant increases of RBC-derived proteins in macrophages after EP, these proteins were rapidly targeted by irreversible oxidation within 1 h. A key example was the peroxidation of Cys94 of hemoglobin-β, which participates in recycling oxidized thiols of peroxiredoxins in mature RBCs and is progressively oxidized in RBCs during aging *in vivo* and *in vitro* (Harper et al., 2015; Wither et al., 2016). Irreversible oxidation of hemoglobin-β Cys94 and of active-site Cys152 of GAPDH suggests that RBC-derived enzymes are rapidly inactivated upon EP. Since Cys152 oxidation of GAPDH is required for catalysis, redox modulation of this residue is sufficient for mediating a metabolic switch from glycolysis to the PPP by limiting fluxes downstream of glyceraldehyde 3-phosphate (Reisz et al., 2016, 2018). Because no significant increases in late glycolysis and lactate production were observed in BMDMs following EP, we cannot exclude the possibility that the redox changes detected of the reactive thiol of Cys152 also targeted the endogenous (macrophage) enzyme. In RBCs, GAPDH membrane localization is associated with binding to the N-terminus of band 3, inhibiting glycolysis to promote PPP flux in response to oxidant challenges (Puchulu-Campanella et al., 2013), whereas subcellular GAPDH relocation in macrophages plays unexpected moonlighting functions (Chauhan et al., 2017). Clustering of band 3 can prime RBC clearance, since they are recognized by natural antibodies of the IgG isotype, triggering complement activation via the classical pathway and enhancement of EP via macrophage complement receptors 1 (CD35) and 3 (CD11b/CD18), and Fc receptors (Lutz, 2004; Arese et al., 2005; Kay, 2005; Klei et al., 2017). Macrophages exposed to increased levels of iron then relocate GAPDH to the membrane, where it interacts with transferrin and serves as a *de facto* transferrin or plasminogen receptor to regulate iron uptake and fibrinolysis (Polati et al., 2012; Chauhan et al., 2017). When not engaged in glycolysis, GAPDH can bind TNF-α mRNA in monocytes to repress the production of this inflammatory cytokine post-transcriptionally (Van den Bossche et al., 2017). Interestingly, though the mechanism that regulates the metabolic shift to the PPP in the phagocytic cell is not better defined in this study, the steady state data and tracing experiments are suggestive of a potential bottleneck downstream to fructose 1,6-bisphosphate at the level of the glycolytic enzyme aldolase. Though speculative at this stage, it is interesting to note that the N-term of RBC-specific band 3 also binds to and inhibits aldolase (other than GAPDH), suggestive that the proteolysis-derived components of the phagocytosed RBC could cross-regulate the metabolism of the macrophage.

Krebs cycle rewiring has profound implications for macrophage effector function and is intimately involved in the remodeling necessary for supporting biosynthetic and bioenergetic requirements (Ryan and O’Neill, 2017). This pathway is disrupted during macrophage activation by inflammatory stimuli and leads to the accumulation of various metabolites, including succinate and citrate (Jha et al., 2015; Lampropoulou et al., 2016; Ryan and O’Neill, 2017; Williams and O’Neill, 2018; Yu et al., 2019). Although succinate and itaconate levels were not significantly elevated before or after EP, a significant SUCC/ITA ratio (*p* = 0.0215) was observed in BMDMs following RBC ingestion, suggesting that the balance between these two metabolites is regulated by EP. Notably, the lack of succinate accumulation following EP is consistent with inhibition of proinflammatory cascades (i.e., stabilization of HIF1α impairs production of downstream targets, such as IL-1β; Selak et al., 2005; Tannahill et al., 2013). Other metabolites that influence prolyl hydroxylases to regulate HIF1α stability include αKG (the precursor to succinate) and fumarate (Boulahbel et al., 2009). While fumarate levels did not increase significantly following EP, αKG, and 2HG (a reversible derivative of αKG) accumulated significantly. Notably, αKG also plays a critical role in epigenetic reprogramming of macrophages (Liu et al., 2017; e.g., it drives Jumonji-Domain Containing Protein 3-dependent epigenetic changes to modulate macrophage effector function) (Diskin and Palsson-McDermott, 2018) whereas, 2HG impairs the activity of αKG-dependent dioxygenases associated with important cellular pathways, such as DNA repair (Ye et al., 2018).

In inflammatory macrophages, HIF1α stabilization can also be achieved via S-nitrosylation mediated by NO availability (Olson and van der Vliet, 2011). But in M2 macrophages, NOS-mediated NO synthesis is prevented by arginine consumption by enzymes such as Arg-1 (Rath et al., 2014). Consistent with increased arginase activity at the expense of NOS activity, EP significantly increased arginine and ornithine levels, but not citrulline. Since RBCs carry both NOS and arginase (D’Alessandro et al., 2019), it is possible that these enzymes could interfere with these pathways in macrophages upon EP. However, NOS is more redox sensitive (Forstermann and Sessa, 2012) and is readily inactivated by EP-induced oxidant stress. Future tracing experiments with ^13^C^15^N-arginine in an RBC-specific arginase knockout mouse could test this hypothesis.

Macrophages that have undergone EP are exposed to the cytosolic and membrane lipidome of the opsonized RBCs. The M2 metabolic phenotype is associated with an increased reliance on mitochondrial metabolism and fatty acid catabolism (Nomura et al., 2016; Remmerie and Scott, 2018). Fatty acid oxidation (FAO) enables subsequent conversion of mitochondrial fatty acids into numerous products, such as acetyl-coenzyme A (acetyl-CoA), NADH, and FADH_2_, which can be used by the cell to generate energy (Remmerie and Scott, 2018). EP promoted consumption of CoA precursors (e.g., pantothenol phosphate) and accumulation of free fatty acids in the absence of significant acyl-conjugated carnitine accumulation. Heme catabolism leads to the activation of anti-inflammatory cascades (e.g., HO-1 activation; Naito et al., 2014), but can also prompt the generation of proinflammatory lipid mediators. Significant increases in intracellular heme catabolites, such as biliverdin (BILV, *p* = 0.0151), following EP confirmed activation of heme catabolism. Further, several metabolites involved in ARA and LA metabolism increased significantly, which could be aided by various hemoproteins (e.g., COXs). During the inflammatory phase following injury or infection, macrophages are natural reservoirs of COX-2 and prostaglandins, and this pathway is, indeed, activated by iron-triggered ferroptotic cascades in response to iron overload in macrophages following phagocytosis of transfused, storage-damaged RBCs (Youssef et al., 2018). Consistent with this, EP-induced increases of specific oxylipins (i.e., prostaglandins, HETEs, diOMEs, and oxoODEs, but not leukotrienes or HODEs). It is intriguing to speculate whether pathological EP, in the context of chronic inflammatory diseases and aging (e.g., in the context of inflammaging-induced anemia; Ferrucci and Balducci, 2008), may excessively activate ferroptotic cascades, thereby exacerbating the risk of bacterial infection. Further, this may be clinically relevant for transfusion recipients of stored RBCs, since REM-mediated clearance is expected to be enhanced by the RBC “storage lesion” (e.g., increased PS exposure, decreased exposure of CD47 (a “do not eat me” signal; Burger et al., 2012a, b), and decreased deformability) (D’Alessandro et al., 2015; Takimoto et al., 2019). This is significant if one considers that ∼25% of transfused RBCs can be cleared within 24 h post-transfusion and there are ∼5 million transfusion recipients annually in the United States.

Most immunometabolism studies to date rely on LPS or cytokine-dependent stimulation of macrophages, and the literature describing the metabolic impact of phagocytosis itself is scarce to non-existent. Thus, this study provides the first metabolic description of macrophages following ingestion of opsonized RBCs. Before summarizing our main conclusions, we would like to highlight some of the limitations of this study: first of all, the *ex vivo* nature of the study and the use of mouse BMDMs may not necessarily recapitulate the phenomenon of EP *in vivo* by tissue-specific residential macrophages in mice or humans; in addition, the lack of potentially relevant controls (e.g., phagocytosis of IgG-coated latex beads or C3b complement-mediated phagocytosis) or the use of other mouse strains than C57BL/6 mice may have differentially impacted our findings, at least to the extent different mouse strains have been reported to differ in iron reduction and homeostasis (Howie et al., 2019) – a key component of EP (Youssef et al., 2018). Acknowledging these limitations, here we report that EP induces changes in the macrophage metabolome that are consistent with polarization toward an M2 metabolic phenotype. One caveat is that these experiments focus on a metabolomic assessment of BMDMs ingesting IgG-opsonized RBCs, or being incubated with IgG, *in vitro*; thus, transcriptional and flow cytometric data from experiments performed *in vivo* are necessary to confirm the apparent upregulation and/or downregulation of the gene products identified from our proteomic analysis. Additionally, no direct interventions were tested to determine whether genetic or pharmacological manipulation could identify which specific, EP-modulated, metabolic pathways are critical for macrophage reprogramming toward a M2 metabolic phenotype. Indeed, the specific pathways identified differ when macrophages ingest other particles (i.e., IgG-opsonized bacteria, viruses, or apoptotic cells) or when Fcγ-receptors are ligated by immune complexes. However, we do show that incubation of BMDMs with IgG alone does not induce metabolic reprogramming similar to that mediated by EP. Future studies are necessary to confirm these results in more physiologically relevant contexts *in vivo*, such as during hemolytic transfusion reactions or autoimmune hemolytic anemia, or in settings of splenic (and hepatic) REM ingestion of senescent RBCs, malaria-infected RBCs, or transfused storage-damaged RBCs.

## Data Availability Statement

The proteomic data has been deposited into the Proteome XChange database (accession: PXD017788).

## Ethics Statement

All procedures were approved by the Institutional Animal Care and Use Committee at Columbia University (New York City, NY, United States).

## Author Contributions

AD’A, SS, LY, and AC conceived and designed the metabolomics studies. AC performed the metabolic analyses of all macrophage experiments. LY performed the *in vitro* EP assay and U-^13^C_6_-glucose tracing experiments with the macrophages. AD’A contributed to the metabolic analysis of the tracing experiments. MD performed the proteomics analysis, and AD’A, JR, AC, and KCH contributed to the analysis of the proteomic data. NP and CM performed the *in vitro* IgG assay with the macrophages. AC and AD’A drafted the first version of the manuscript, and AC, JR, and AD’A prepared all figures. All co-authors contributed to preparing the final manuscript.

## Conflict of Interest

Although unrelated to the contents of the manuscript, the authors declare that AD’A and KCH were founders of Omix Technologies Inc. and Altis Biosciences LLC. AD’A and SS were consultants for Hemanext, Inc. SS was a consultant for Tioma, Inc., and was the Executive Director of the Worldwide Initiative for Rh Disease Eradication (WIRhE). The remaining authors declare that the research was conducted in the absence of any commercial or financial relationships that could be construed as a potential conflict of interest.
