# Supplementary material for: Metabolic Reprogramming of Mouse Bone Marrow Derived Macrophages Following Erythrophagocytosis
Source: Front Physiol. 2020 Apr 30;11:396. doi: 10.3389/fphys.2020.00396 (PMC7204509; doi:10.3389/fphys.2020.00396)
Supplement: Supplementary file 2 [file Table_1.docx]

**Metabolic Reprogramming of Bone Marrow Derived Macrophages Following Erythrophagocytosis**

Supplementary Material


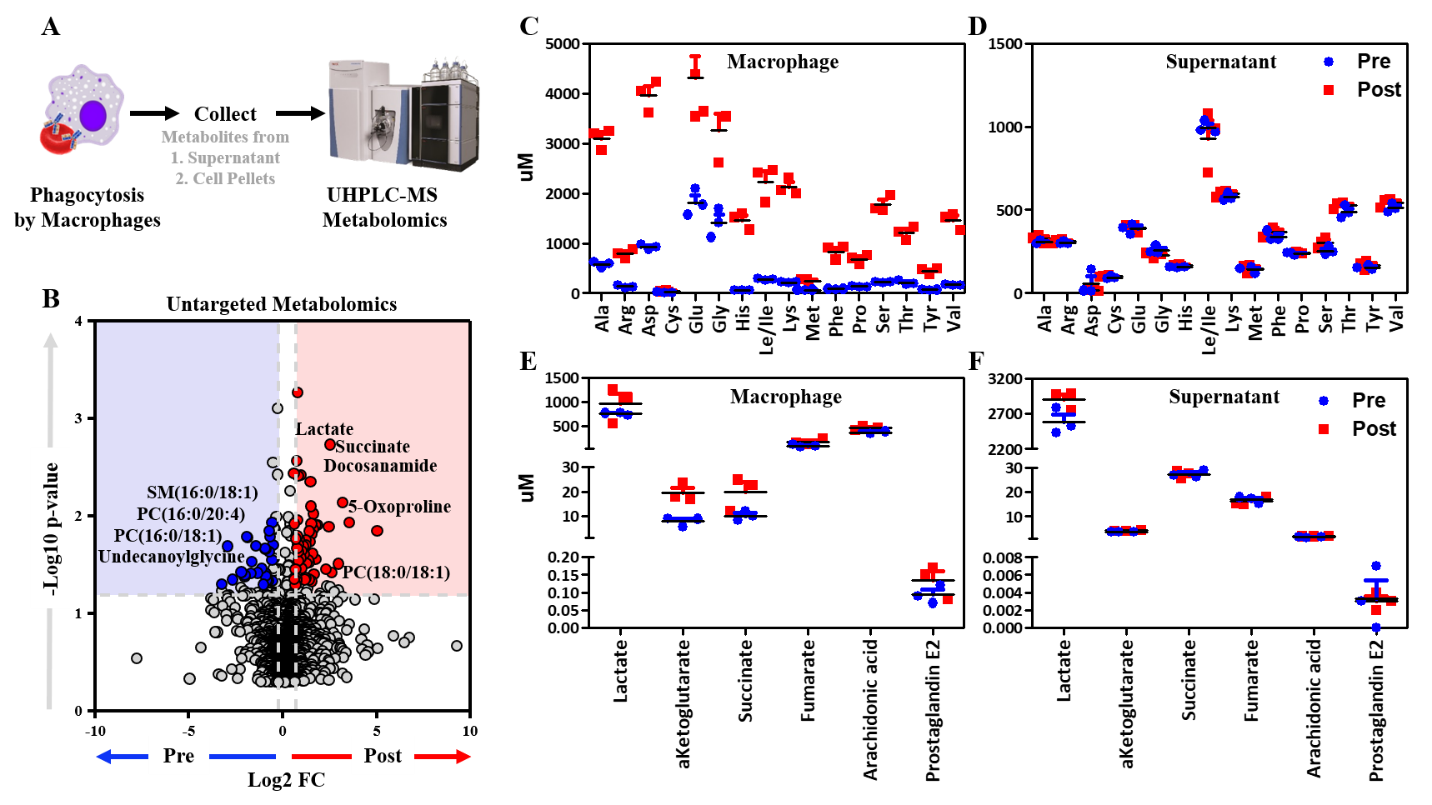


**Supplementary Figure 1: Assessment of BMDM phagocytosis.** Mouse RBCs were opsonized with IgG and incubated with BMDMs in vitro. Metabolites extracted from the extracellular media (supernatant) and cell pellets were then analyzed by UHPLC-MS. **(A)** Univariate analysis of the BMDM metabolome using untargeted metabolomics to identify metabolites that change significantly due to EP in cell extracts **(B)**. The region highlighted in red (FC ≥ 2.0; *p*-value < 0.05) indicates metabolites present in significantly higher amounts in BMDMs after EP (Post); whereas, the region highlighted in blue (FC ≤ 0.5; *p*-value < 0.05) indicates metabolites found to be accumulated in BMDMs before EP (Pre). Amino acid metabolism in BMDM cells **(C)** and supernatants **(D)**. General metabolic survey of key pathways from BMDM extracts, comparing similar metabolites but found in the cell **(E)** or extracellular medium **(F)**. In these plots (C-F), the y-axis represents the concentration of metabolite measured (μM) by absolute quantitation based on known concentrations of internal standards.

**
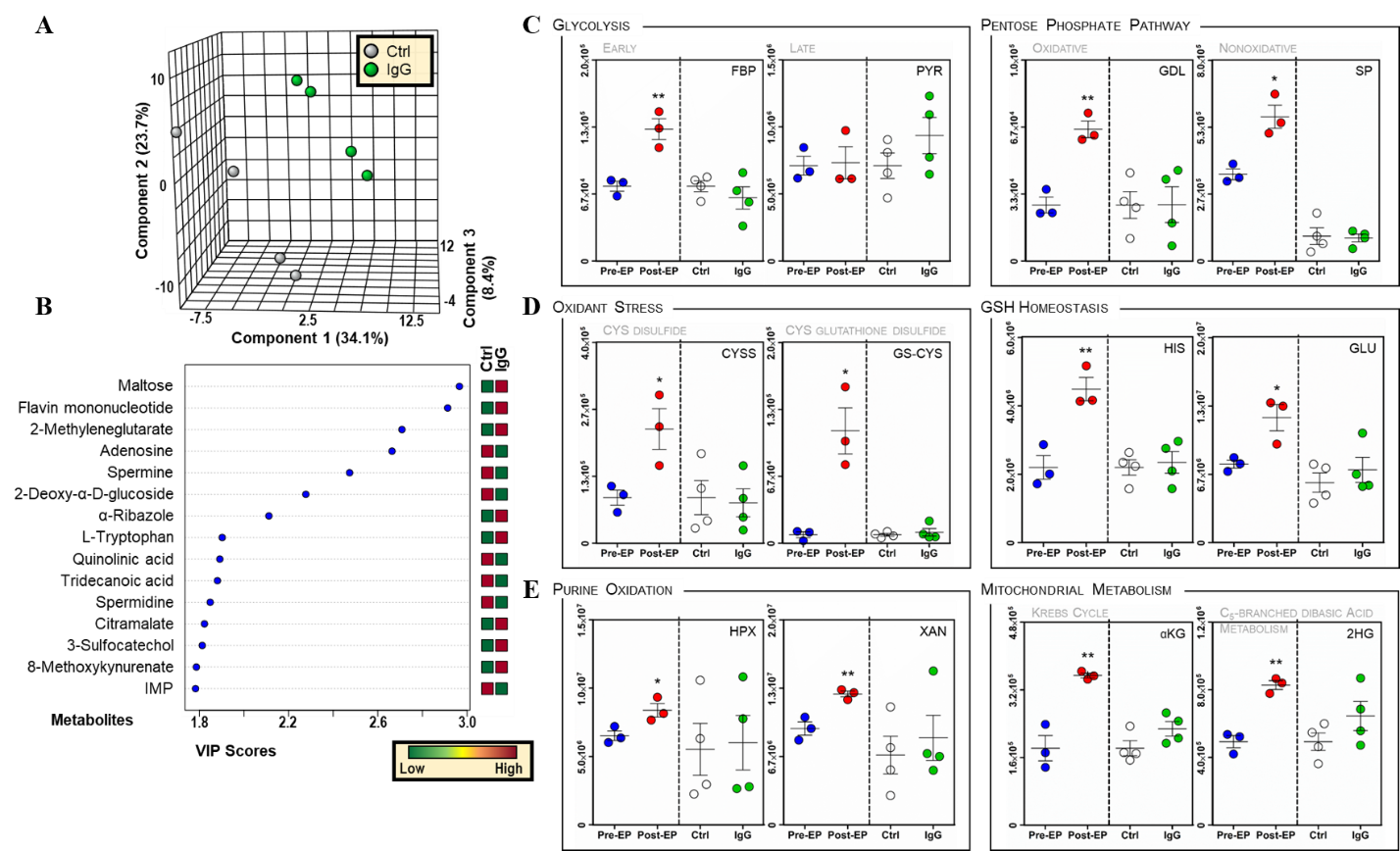
**

**Supplementary Figure 2: The BMDM metabolome is differentially reprogrammed by EP, as compared to incubation with IgG alone.** Metabolic profiles were sufficient to discriminate between BMDMs that had been incubated with PBS (Ctrl, *white*) or with antibody alone (IgG, *green*), as demonstrated by partial least squares-discriminant analysis (PLS-DA) **(A)**. The PLS-DA variable importance in projection (VIP) plot reveals the top 15 significant metabolites that were identified in BMDMs that had been incubated with IgG **(B)**. VIP scores were calculated for each metabolite (*blue circles*) and ranked in the context phagocytosis (Ctrl/IgG) (high score, *red*; low score, *green*). Comparisons were performed to BMDMs before and after EP (Pre-EP, *blue*; Post-EP, *red*) for particular key pathways, including glycolysis and the pentose phosphate pathway **(C)**, oxidant stress and GSH homeostasis **(D)**, and purine oxidation and mitochondrial metabolism **(E)**. For all plots (C-E), the y-axis represents relative intensity (a.u.), and data scaling was performed based on control groups to make metabolic features comparable between the *in vitro* EP and IgG assays. **p* ≤ 0.05; ***p* ≤ 0.01; ****p* ≤ 0.001 (unpaired t test, 2-tailed distribution)


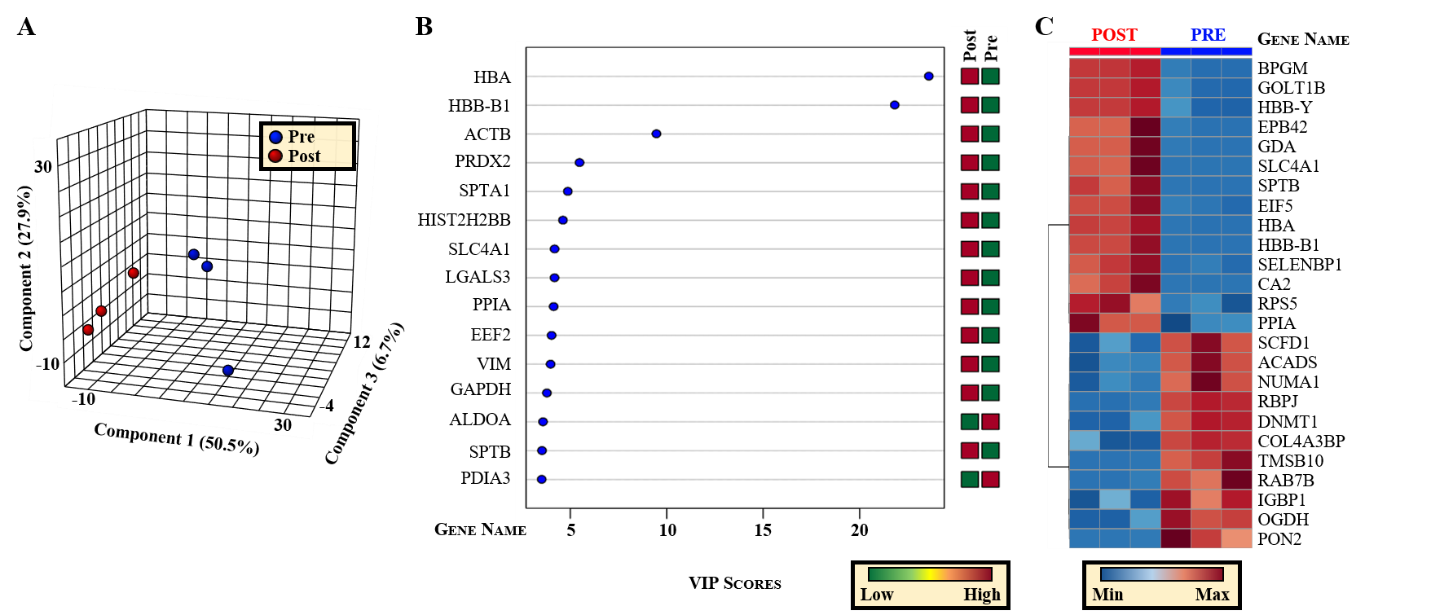


**Supplementary Figure 3: Multivariate analysis reveals extensive changes to the BMDM proteome due to EP.** Changes to the BMDM proteome were sufficient to discriminate between BMDMs before (Pre, *blue*) and after EP (Post, *red*), as demonstrated by PLS-DA **(A)**. PLS-DA VIP plot reveals the top 15 significant proteins in BMDMs that were affected by EP **(B)**. VIP scores were calculated for each protein (listed by gene name, *blue circles*) and ranked in the context of EP (Post/Pre) (high score, *red*; low score, *green*). The top 25 proteins found to be significantly altered by EP in BMDMs are plotted as a hierarchically-clustered heat map based on *p*-value **(C)**.
